# Perspective: Mitochondria-ER Contacts in Metabolic Cellular Stress Assessed by Microscopy

**DOI:** 10.3390/cells8010005

**Published:** 2018-12-21

**Authors:** Alessandra Stacchiotti, Gaia Favero, Antonio Lavazza, Raquel Garcia-Gomez, Maria Monsalve, Rita Rezzani

**Affiliations:** 1 Anatomy and Physiopathology Division, Department of Clinical and Experimental Sciences, University of Brescia, Viale Europa 11, 25123 Brescia, Italy; gaia.favero@unibs.it (G.F.); rita.rezzani@unibs.it (R.R.); 2 Interdipartimental University Center of Research “Adaptation and Regeneration of Tissues and Organs-(ARTO)”, University of Brescia, 25123 Brescia, Italy; 3 ANZAC Research Institute, Concord Hospital, NSW 2139 Sydney, Australia; 4 Istituto Zooprofilattico Sperimentale della Lombardia ed Emilia Romagna-IZSLER, 25124 Brescia, Italy; antonio.lavazza@izsler.it; 5 Instituto de Investigaciones Biomedicas “Alberto Sols” (CSIC-UAM), 28029 Madrid, Spain; rgarcia@iib.uam.es (R.G.-G.); mpmonsalve@iib.uam.es (M.M.)

**Keywords:** mitochondria, endoplasmic reticulum, MERCs, MAMs, obesity, neurodegenerative diseases, microscopy

## Abstract

The interplay of mitochondria with the endoplasmic reticulum and their connections, called mitochondria-ER contacts (MERCs) or mitochondria-associated ER membranes (MAMs), are crucial hubs in cellular stress. These sites are essential for the passage of calcium ions, reactive oxygen species delivery, the sorting of lipids in whole-body metabolism. In this perspective article, we focus on microscopic evidences of the pivotal role of MERCs/MAMs and their changes in metabolic diseases, like obesity, diabetes, and neurodegeneration.

## 1. “Contactology” a New Branch of Cytology

“Contactology” is a recent term proposed by Csordas et al. [1] to indicate the study of the physical and functional bridge between mitochondria and endoplasmic reticulum (ER), and it represents a sort of “synapse” that drives cellular physiology. The nanometric distance that separates endoplasmic reticulum tubules and the outer mitochondrial membrane is a dynamic essential passage of lipids, glycogen, and calcium ions essential for cellular activity and disrupted in diseases [2,3,4,5,6]. Despite recent in-depth authoritative reviews on its molecular biology and biochemistry [7,8], the detailed visualization of this close connection is still a challenge. Multiple microscopic methods are required to best characterize this structure at the nanometric level and to quantify the membrane contacts in different experimental and pathological conditions. This perspective article intends to provide researchers with an updated insight on imaging of the interplay between mitochondria and the ER in metabolic stress, like diabetes and obesity, and in neurodegenerative diseases.

The use of microscopy to visualize the spatial arrangements of eukaryotic cells and their subcellular organelles is a fundamental tool for acquiring biological and medical knowledge. Here, we provide a brief history of the significant milestones that have contributed to our present day understanding of mitochondria and the endoplasmic reticulum (ER). This timeline is closely associated with the evolution of microscopy. The word “mitochondria” was first used at the end of the nineteenth century by Carl Benda, a German microbiologist, who observed filamentous structures inside cells that were stained by crystal violet under a simple light microscope. In the 1950s, mitochondrial morphology and oxidative function became clearly defined [9,10]. In 1953, ultrastructural images of mitochondria were published independently by Sjostrand [11] and Palade [12]. In these seminal manuscripts, the authors characterized the outer and inner membranes and defined “cristae” as the arrangements in the matrix. More recently, high voltage electron microscopes and computerized reconstruction of tilt series images have led to the three-dimensional (3D) visualization of mitochondria [13,14]. Using electron tomography, we can now appreciate cristae junctions, a sub-compartment of mitochondrial cristae that are involved in the exchange of lipid and proteins and their alterations in diseases [15]. It has been shown that mitochondrial cristae are affected in neurological diseases like Alzheimer’s and Parkinson’s or amyotrophic lateral sclerosis [16,17,18]. Moreover, the dogma that each cell has a fixed number of cristae in its mitochondria has been recently challenged. Further to this, the leg muscles of athletes with long term endurance training display a higher number of cristae in mitochondria, in contrast to sedentary subjects, which might contribute to an increase in performance [19]. Recent work has shown that cristae morphology is coupled with higher oxidative phosphorylation and that increased cristae appear to create more surface area for adenosine triphosphate (ATP) synthesis [20]. Abnormal cristae and smaller mitochondria have been observed during apoptosis, when cytochrome c is released from mitochondria into the cytoplasm [21]. Further, the shape and localization of mitochondria differ by cell type. For example, in cardiomyocytes, there are different populations of mitochondria localized at the subsarcolemmal and intermyofibrillar area [22], whereas in renal proximal tubular cells the mitochondria appear to be predominantly elongated [23] and neurons display a heterogeneous distribution of mitochondria that appear to be linked to calcium flux [24]. Mitochondria shape and dimensions are known to adapt and shift greatly under metabolic stress conditions, like oxidative damage, hypoxia, and glycemic changes [25,26]. “Fusion” is the process that produces elongated or tubular mitochondria, whereas “fission” is the shortening process, and, interestingly, both processes may occur in the same mitochondrion under different metabolic states [27]. Recently, it has been shown that mild upregulation of the protein optic atrophy 1 (OPA 1) in the inner mitochondrial membrane reversed metabolic stress and apoptosis and ameliorated cristae number in fibroblasts and hepatocytes [28].

Another important discovery in the history of microscopy was made by Palade, a Romanian biologist, who visualized electron-dense particles that are associated with endoplasmic cisternae, known at that time as “Palade particles”, but known now as ribosomes [29]. Palade was awarded the Nobel Prize in Physiology and Medicine in 1974 for his work describing the smooth (SER) and rough endoplasmic reticulum (RER) membranes with or without ribosomes [30]. To date, the ER, as defined by modern imaging techniques, is known to be composed of perinuclear sheets (RER) and peripheral tubules (SER) that are localized to different cytoplasmic compartments [31,32]. In COS-7 cells the ER subdomains, called organized smooth endothelial reticulum (OSER), have been visualized by ultra-microscopy [33]. Similar features have been detected in neurons that were taken from patients with amyotrophic lateral sclerosis [34]. Three-dimensional imaging has further characterized the shape and extent of ER in mammalian cells and demonstrated the importance of ER morphology and its involvement in processes, like calcium flux, unfolded protein response, and cell death [35,36].

The dynamic nature of mitochondria and the ER has led scientists to consider the alternative hypothesis of a cytoplasmic environment as one of the non-static interconnected organelles [37,38,39]. Csordas et al. [40] demonstrated, by electron microscopy, the close proximity between mitochondria and ER and then calculated that this distance corresponded to 25–40 nm for RER-mitochondria and 10 nm for SER-mitochondria. Examples of juxtaposition, without the loss of organelle integrity, of the outer mitochondrial membrane and ER were shown in this seminal work [41], and interestingly, ER enclosing mitochondria in specific sites contributed to their fission [42].

The nomenclature of the peculiar arrangement between mitochondria and ER is often confused due to the different techniques adopted in various laboratories to study these components. Thus, in mammalian cells, mitochondria-ER juxtaposition was defined as mitochondria-ER contacts (MERCs) [43] or membrane contact sites (MCSs) [44], but also mitochondria-associated ER membranes (MAMs), [45,46,47]; while in mutant yeast, the same observation is known as a ER-mitochondria encounter structure (ERMES) [48,49].

## 2. Imaging of Mitochondria-ER Interface by Electron Microscopy

The exchange of lipids is essential for the maintenance of mitochondrial membranes and it is driven at the ER-mitochondria juxtaposition [50]. In particular, sterols, such as phosphatidylserine and phosphatidylcholine that are produced in the ER, are transported into mitochondria [51]. Recent studies demonstrated that a reduction in phosphatidylethanolamine (PE) in PSB-2 cells, a mutant CHO cell line, induced abnormal MAMs and led to aberrant mitochondrial fragmentation [52]. Moreover, in cells, PE is anchored to autophagosomes in a process called lipidation and this anchor is necessary for efficient autophagy [53]. Remarkably, as observed by conventional and immunogold electron microscopy, autophagosomes were also produced at MAMs in starved COS-7 cells [54].

**Figure 1:**
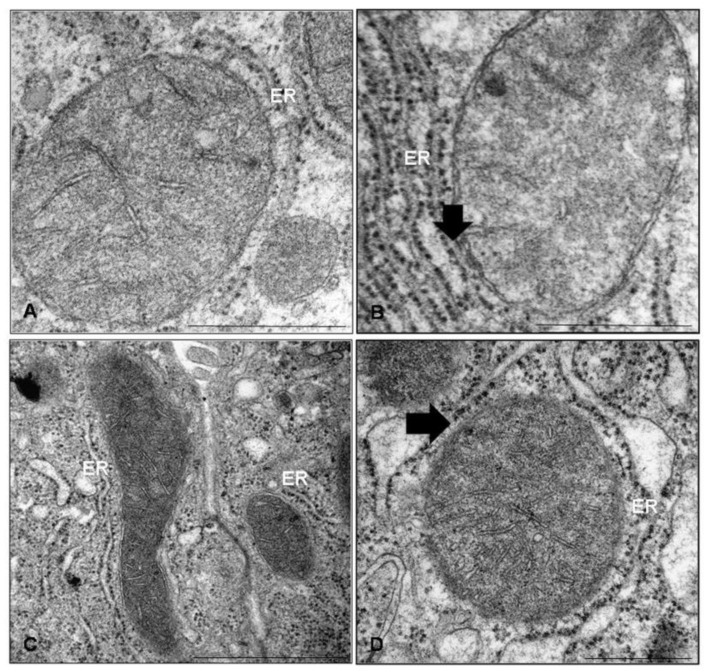
Mitochondria-associated endoplasmic reticulum membranes (MAMs) ultrastructure in adult mice liver changed with nutrition. (**A**) C57BL6/J mice liver fed a maintenance diet; (**B**) leptin-deficient obese mice presented abnormal endoplasmic reticulum (ER) cisternae and narrowing of contacts; (**C**) repeated starved C57BL6/J mice showed regular distance and elongated mitochondria; (**D**) repeated starved sirtuin 1 heterozygous mice presented close ER-mitochondria contact and swollen cisternae. (ER): Endoplasmic reticulum; arrows identify MAM narrowing. Scale bar: A, B, D 500 nm; C 1 µm.

In HT1080 fibrosarcoma cells, the distance between SER-mitochondria and RER-mitochondria is 8 nm and 50–60 nm, respectively [55]. In mice liver that was analyzed under cryo-electron microscopy and tomography, MERCs/MAMs length and thickness changed upon metabolic energy intake, i.e., increased under starvation but decreased in over-nutrition [43,56]. It has been estimated that, in mammalian cells, about 20% of the mitochondria surface is attached to the ER membranes [57]. Moreover, during early phases of ER stress response, HeLa cells that were treated with tunicamycin enhanced the ER-mitochondria contacts to sustain mitochondrial metabolic activity [58]. Arruda et al. [59] demonstrated enriched MAMs in the liver of leptin-deficient mice and in dietary induced obese mice. This finding was related to an excessive calcium flux from the ER to mitochondria and consequent apoptosis. Our research group reported that melatonin supplementation restored the enlargement of MAMs distance in leptin deficient mice liver as compared to its abnormal narrowing in obese mice [60]. This gap was evident in C57BL6/J mice liver, but decreased in leptin deficient mice, together with ER fragmentation and altered mitochondria (Figure 1A,B). Moreover, caloric restriction of transgenic mice that are heterozygous for sirtuin 1, a class III histone deacetylase crucial for mitochondrial health [61], led to excessive MAMs contacts in hepatocytes when compared to wild type mice (Figure 1C,D). The maintenance of a regular MAMs distance was critical for insulin signaling and affected insulin resistance [62]. Indeed, in cyclophilin D KO mice liver, the absence of this mitochondrial protein resident in the MAM interface blocked insulin flux and its local response. Disrupted MAMs have also been characterized in neurodegenerative disorders, including Alzheimer’s and Parkinson’s disease [63].

Furthermore, among the powerful imaging techniques of the brain, we must consider scanning electron microscopy, which is able to provide multiple 3D reconstructions of membrane contact sites in neurons by a focused ion beam [64].

## 3. Imaging of Mitochondria-ER Interface by Dynamic Fluorescent Light Microscopy

The major drawback of electron microscopy is the necessity of fixation that makes it impossible to follow dynamic events. Moreover, the nanometric distance between outer mitochondrial membrane and ER represents a challenge for conventional optical microscopy, because it is below the resolution limit. Currently, confocal and super resolution microscopy are overcoming these limitations and allowing the visualization of MAMs and calcium flux using fluorescent probes [65]. New methods, like time lapse microscopy and soft-x-ray tomography, have further characterized the 3D structure of MAMs in COS-7 cells [66]. To verify the role and dynamics of MAM resident proteins in vivo, different interesting methods have been developed based on green fluorescent protein (GFP) or Venus yellow fluorescent protein (YP) probes and confocal laser or super-resolution microscopy. One method, called the dimerization dependent assay, employed non-fluorescent monomers; one that was located on the ER and the other on the mitochondrial side of MAMs, which dimerized and became fluorescent when their distance decreased under 10–20 nm [67]. Another confocal technique, called fluorescence resonance energy transfer (FRET) based indicator of ER-mitochondria proximity, followed calcium flux in MAMs in mutated mouse fibroblasts using donor and acceptor probes to produce fluorescence at nanoscale level [68]. Total internal reflection fluorescence (TIRF) microscopy, which is based on a high power laser light, has been successfully used to image the phosphatidylserine (PE) transfer from ER to mitochondria in live HeLa cells in an aqueous medium with lower refractive index [69]. A recent GFP-based fluorescent technique that was developed by Cieri et al. [70] firstly indicated that, in HeLa and HEK293 cells, there were different types of short (8–10 nm) and long (40–50 nm) tethers between mitochondria and ER that responded differently to pharmacological stimuli and starvation. To best detect dynamic changes to MAMs in the liver induced by stressors, like glucose or apoptotic inducers, many laboratories have adopted a powerful fluorescent technique, called “proximity ligation assay” [71]. Using this method and fluorescent analysis of calcium transport, Rieusset et al. [72] have demonstrated that abnormal MAMs and disrupted calcium signaling greatly contributed to insulin resistance in hepatocytes in cyclophilin D knockout mice. Super-resolution microscopy now provides scientists with the ability to follow single fluorescent molecules and their trajectories in the ER in neurons and how their flux is perturbed in neurodegenerative conditions [73,74].

These technological advances now make it feasible to examine the role of MAMs connections in the development of neurodegenerative disease and provide tools for examining whether the reversal of these changes may be harnessed for therapeutic purposes. Synthetic linkers have been successfully tested to restore regular MAMs in metabolically stressed hepatocytes [59] and the overexpression of the neuronal calcium sensor 1, a resident linker, in fibroblasts of the Wolfram syndrome patients restored MAMs and proper calcium transfer to mitochondria [75]. Further refinement of our knowledge through microscopy has provided opportunities to translate these preliminary results into clinical applications.

In conclusion, there is no preferred method to visualize MAMs/MERCs/MCSs in cells and every microscopy technique has advantages and disadvantages that must be considered.

In our opinion, the best approach is to combine different methods. In Table 1, the advantages and disadvantages of MAMs/MERCs/MCSs imaging are summarized.

## Figures and Tables

**Table 1:** MAMs/mitochondria-ER contacts (MERCs)/membrane contact sites (MCSs) imaging in cells.

Microscopy/Methods [References]	Advantages	Disadvantages
Transmission Electron Microscopy-TEM [40,41,42,59,60]	Elective for nanoscale resolution	Not suitable for living cells Technically hard Expensive
Cryo-TEM plus tomography [43]	3D images in small volumes	Not suitable for living cells
Scanning Electron Microscopy-SEM FIB [64]	Good resolution 3D images in large volumes	Not suitable for living cells
Confocal Laser Fluorescence Microscopy [41,48,49,52,56,62,66,70,71,72]	Suitable for living cells and dynamic events Quantification contacts	Toxic for cells after long time Lower Resolution Unstable Specific probes required
Total Internal Reflection Fluorescence Microscopy [69]	High Brightness Suitable for dynamic events	Sensitive to refraction index Thermogenic
Super-resolution Microscopy [67,73,74]	High Brightness 3D images Quantification contacts	Specific probes required Expensive
